# Supplementary material for: Role of NS1 and TLR3 in Pathogenesis and Immunity of WNV
Source: Viruses. 2019 Jul 3;11(7):603. doi: 10.3390/v11070603 (PMC6669597; doi:10.3390/v11070603)
Supplement: Supplementary file 1 [file viruses-11-00603-s001.zip › Supplementary_R1/S2_FileR1.pdf]

Supplementary Data 2

Susceptibility of Black 6 mice against WNV infection

As our previous studies with WNV ITA09 had been performed in a Balb/C mouse model but TLR3KO mice are bred on a C57BL/6 background, we characterized the dose-dependent outcome of WNV infection in C57BL/6 mice. For this purpose, six groups of mice (4 mice per group) were used. Three groups of C57BL/6 mice were administered intraperitoneally (i.p.) with 10-fold dilutions of WNV ITA09, i.e. one group was inoculated with 10^5 pfu per mouse, a second group with 10^4 pfu and a third group with 10^3 pfu of ITA09. As a positive control, one group of Balb/C mice was inoculated with 10^4 pfu ITA09 per mouse, whereas each one group of C57BL/6 and Balb/C, respectively, were used as mock-inoculated (PBS) negative controls. As shown in Fig. S3A, all mock-inoculated mice survived, whereas the WNV-inoculated mice died in a dose-dependent manner. Specifically, all four (100%) C57BL/6 mice infected with 10^5 pfu ITA09 virus died within 10 days after inoculation, whereas only three (75%) mice developed a lethal WNV infection after inoculating 10^4 pfu and only two (50%) after inoculating 10^3 pfu. Moreover, the 10^4 pfu-positive control killed also three of the four (75%) of the Balb/C mice. Thus, the lethal dose of WNV ITA09 was comparable in the two mouse strains.

Interestingly, viremia was detected by quantitative reverse transcription-PCR (qRT-PCR) in the plasma samples of many WNV-inoculated mice, even in those with lower doses of inoculated virus (Fig. S3B). In contrast, non-WNV-inoculated mice remained negative in this assay (all negative values not included in the figure). These data corroborated the successful inoculation of the mice, even of those not succumbing to lethal WNV-infection.
At the end of the experiment, the spleens (Fig. S3C) and brains (Fig. S3D) of the mice were also analyzed for WNV genomes by qRT-PCR. For this purpose, the organs were minced before 10% suspensions of the tissues were prepared for quantitative analysis. Indeed, WNV persisted in the spleens of those animals, which succumbed to a lethal WNV-infection. The median viral load amounted to approximately 10^4 genomes per ml and appeared to be independent of the original dose of inoculation (negative values not included in the figure). In contrast, WNV ITA09 reached the brains of all of the inoculated individuals and the number of viral genomes was consistently close to 10^6 genomes per ml in those animals who succumbed to the lethal WNV-infection, whereas was significantly lower ($p<0.002$) in the surviving individuals (green dots in Fig. S3D).
As the inoculation of C57BL/6 mice with 10^5 pfu WNV ITA09 led to a consistent high-dose infection of the brain, followed by 100% lethality (approximately 100 LD_{50}), we decided to use this dose for the upcoming vaccination-challenge experiments.

Fig. S3. Susceptibility of C57BL/6 mice against WNV (ITA09) infection. Three groups of C57BL/6 mice (n=4 per group) were inoculated with different doses of WNV ITA09 strain. As a positive control, one group of BalbC mice was inoculated with 10^4 pfu of the virus, whereas each one group of C57BL/6 and BalbC, respectively, were mock inoculated with PBS.
A) Survival curve.
B) Quantitative detection (qRT-PCR) of WNV RNA in plasma samples of the inoculated mice on dpi 2 and 4, respectively. PCR-negative animals were excluded from the plot.
C) qRT-PCR of WNV RNA in 10% suspensions of spleen tissue of the mice at the end of the experiment. PCR-negative animals were excluded from the plot.

D) qRT-PCR of WNV RNA in 10% brain suspensions of mice at the end of the experiment. Black dots represent individuals that succumbed to lethal WNV, whereas green dots represent the PCR-positive surviving individuals. PCR-negative animals were excluded from the plot.
B,C,D) individual as well as median values of the PCR-positive animals are shown.

Materials and Methods

Animals, and challenge experiment

C57BL/6J, Balb/c and B6;129S1-Tlr3tm1Flv/J (TLR3 knockout; TLR3KO) female mice were used for animal experiments.
Female wild type (WT) C57BL/6 mice (6-8 weeks old) and BALB/c mice (6-8 weeks old) were purchased from Charles River (Germany) and housed at the Institute of Laboratory Animal Sciences.
For the establishment of WNV model in C57BL/6 mice, 12-14 weeks old female C57BL/6 mice (n=4) were injected intraperitoneally (i.p.) with either 200 µl of 10^3, 10^4 or 10^5 plaque forming units (pfu) of WNV lineage 1 ITA-09 strain (L Barzon et al., 2009). 12-14 weeks old BALB/c mice (n=4) were injected i.p. with 200 µl of 10^4 pfu of the virus as a positive control. Blood samples were collected at day 2 and 4, post-inoculation. Brain and spleen samples were collected after euthanisation. All the samples were tested for viral load using quantitative real time RT PCR.

WNV RNA detection in plasma and tissue samples
For WNV RNA detection in plasma, brain and spleen samples, total nucleic acids were purified from samples by using a MagNA Pure 96 SV nucleic acid purification kit on a MagNA Pure 96 System (Roche, Basel, Switzerland). Real-time RT-PCR targeting the WNV NS5 gene (Linke, Ellerbrok, Niedrig, Nitsche, & Pauli, 2007) was performed using a One Step Real Time kit (Thermo Fisher Scientific, Waltham, MA, USA) on a 7900 HT Sequence Detection System instrument (Thermo Fisher Scientific), as previously reported (Luisa Barzon et al., 2013)
