# Supplementary material for: Role of NS1 and TLR3 in Pathogenesis and Immunity of WNV
Source: Viruses. 2019 Jul 3;11(7):603. doi: 10.3390/v11070603 (PMC6669597; doi:10.3390/v11070603)
Supplement: Supplementary file 1 [file viruses-11-00603-s001.zip › Supplementary_R1/Fig S5R1.pdf]

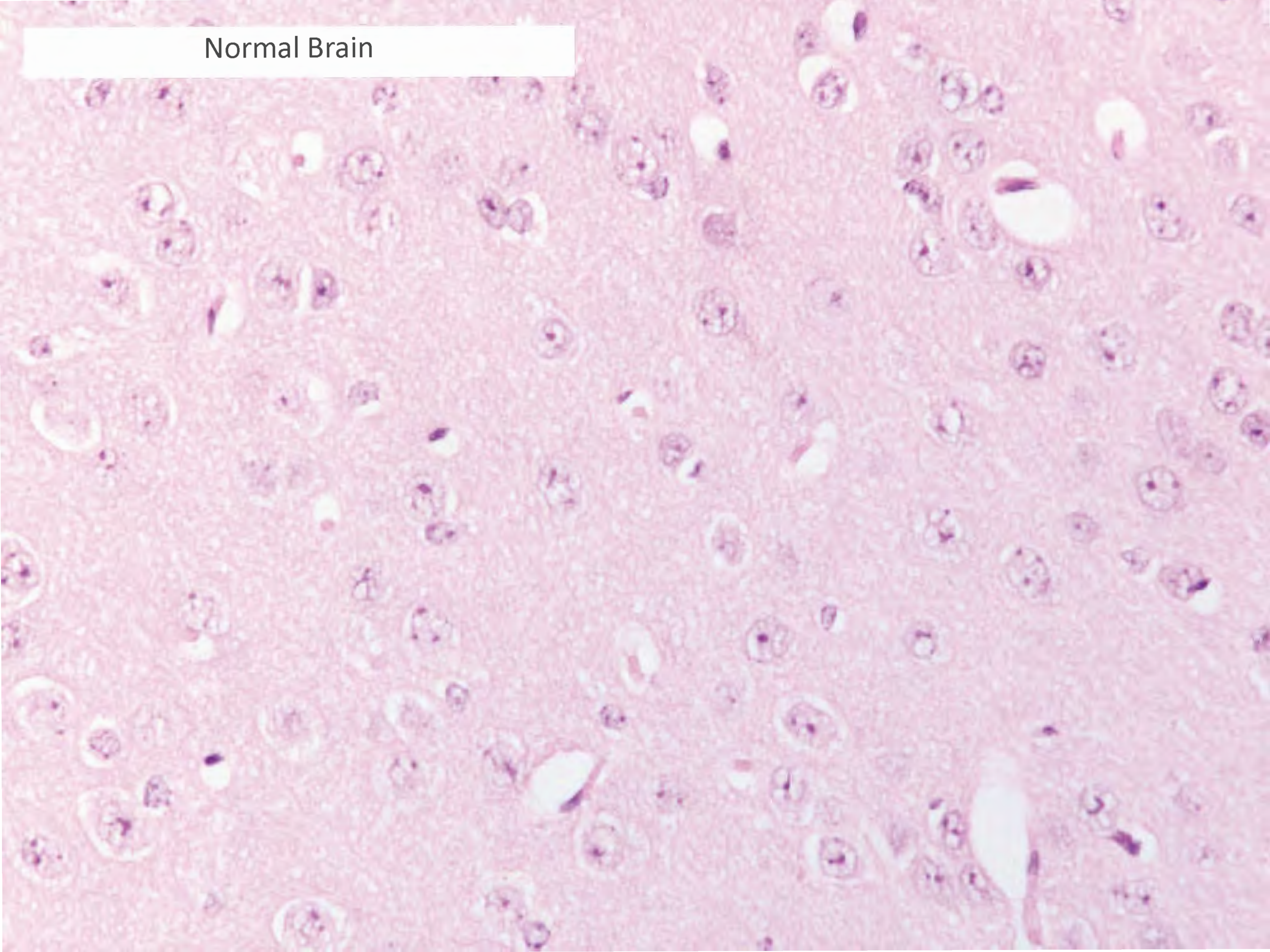
Normal Brain

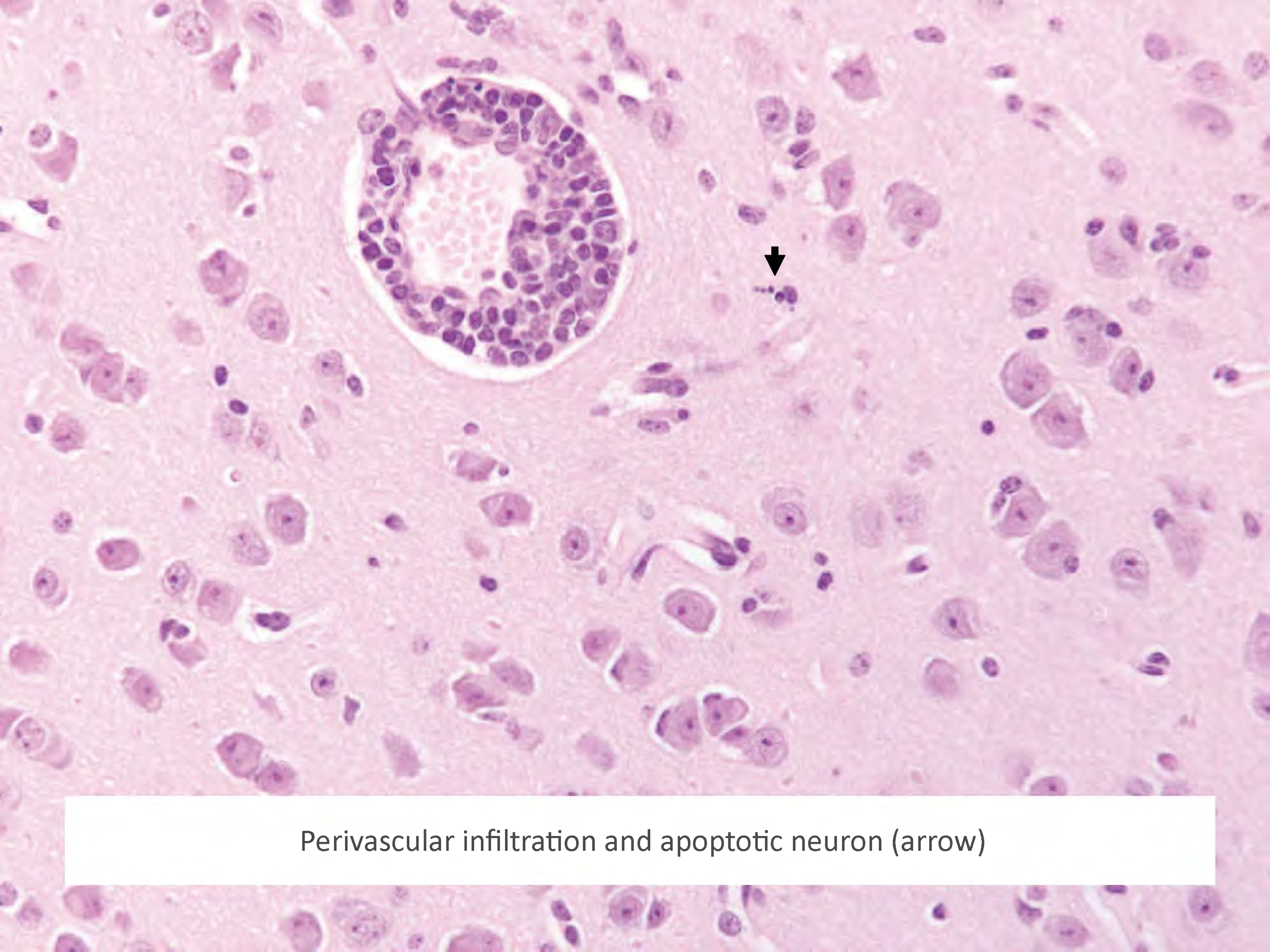

Perivascular infiltration and apoptotic neuron (arrow)

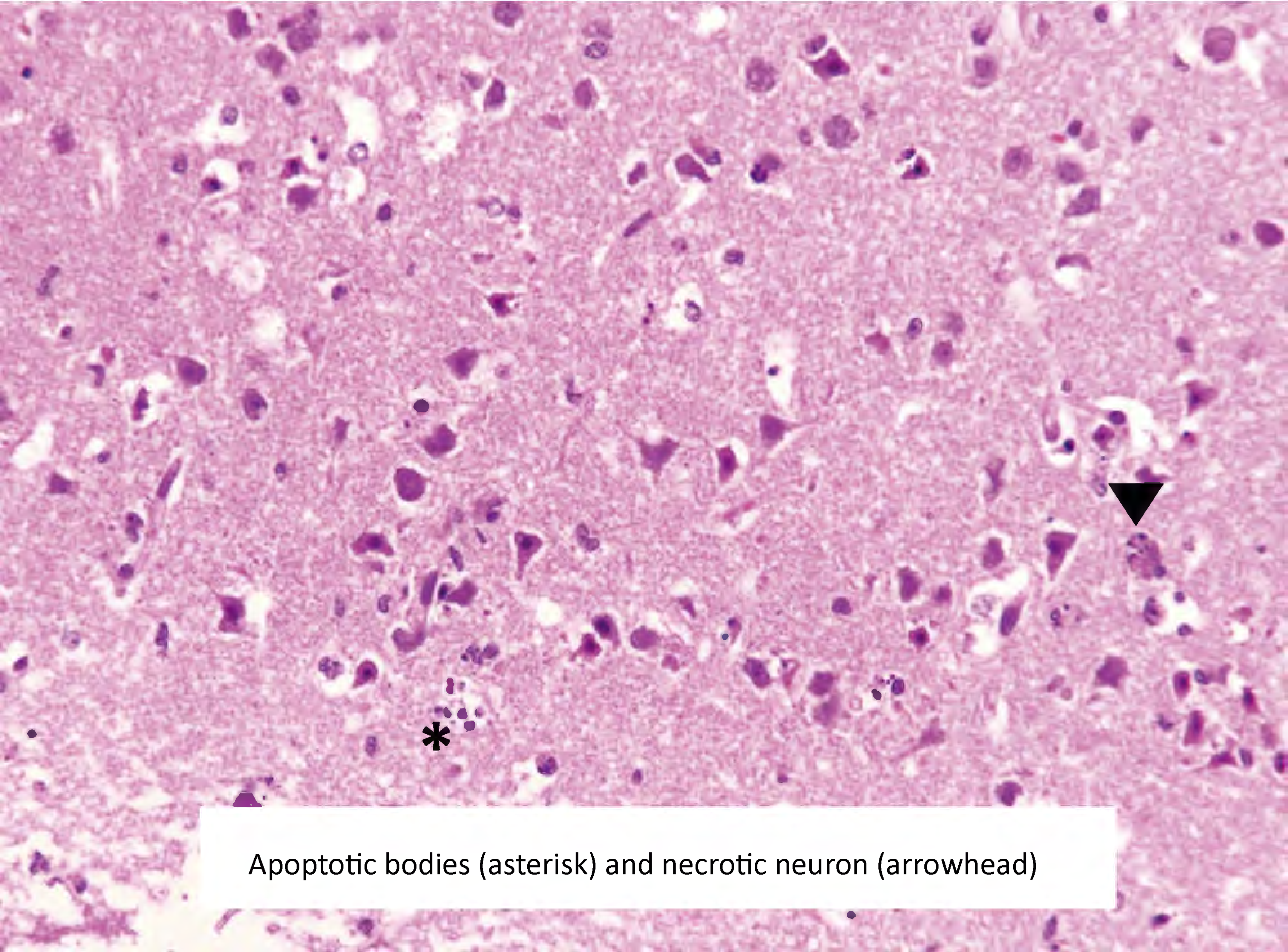

Apoptotic bodies (asterisk) and necrotic neuron (arrowhead)

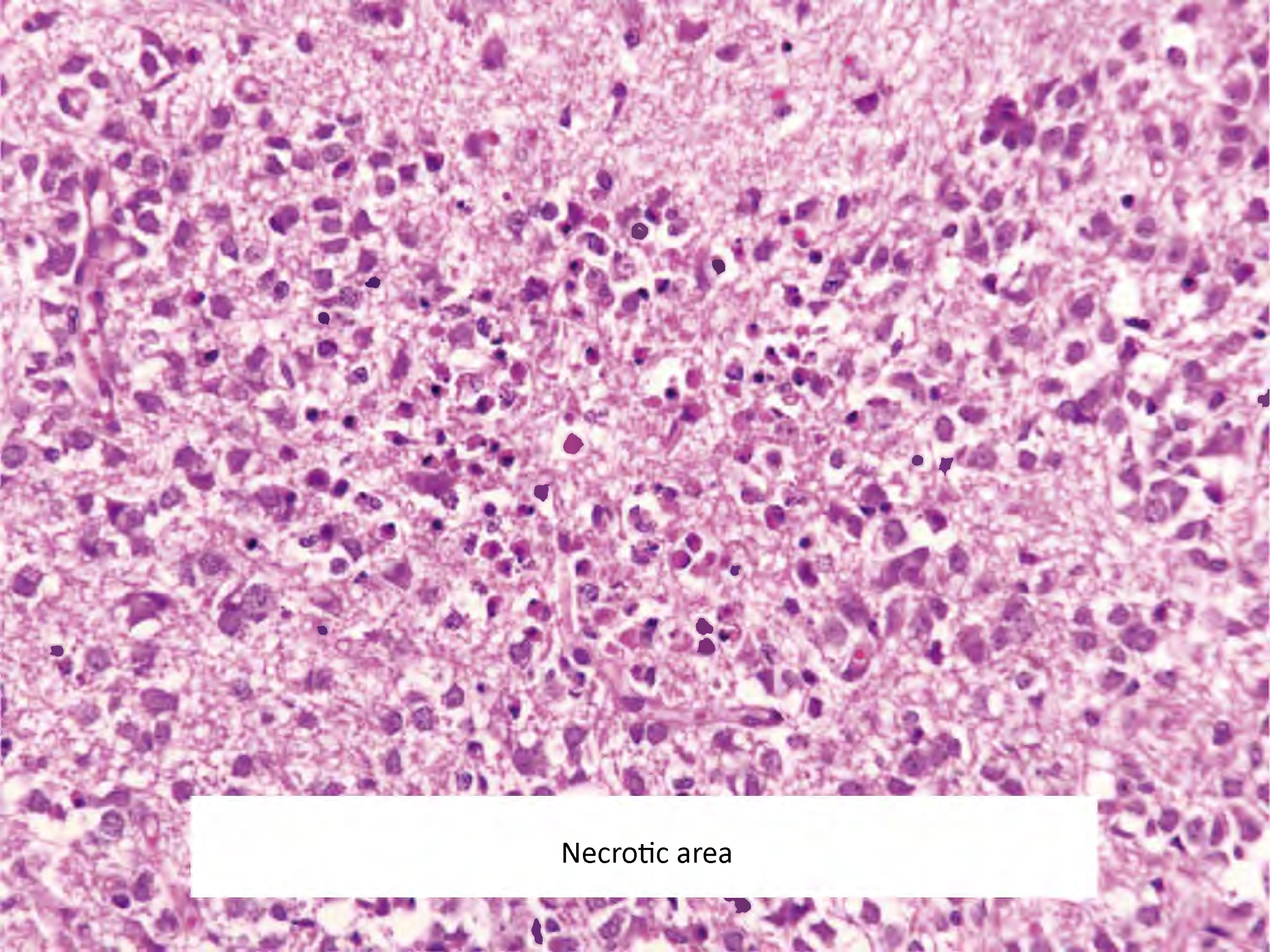

Necrotic area

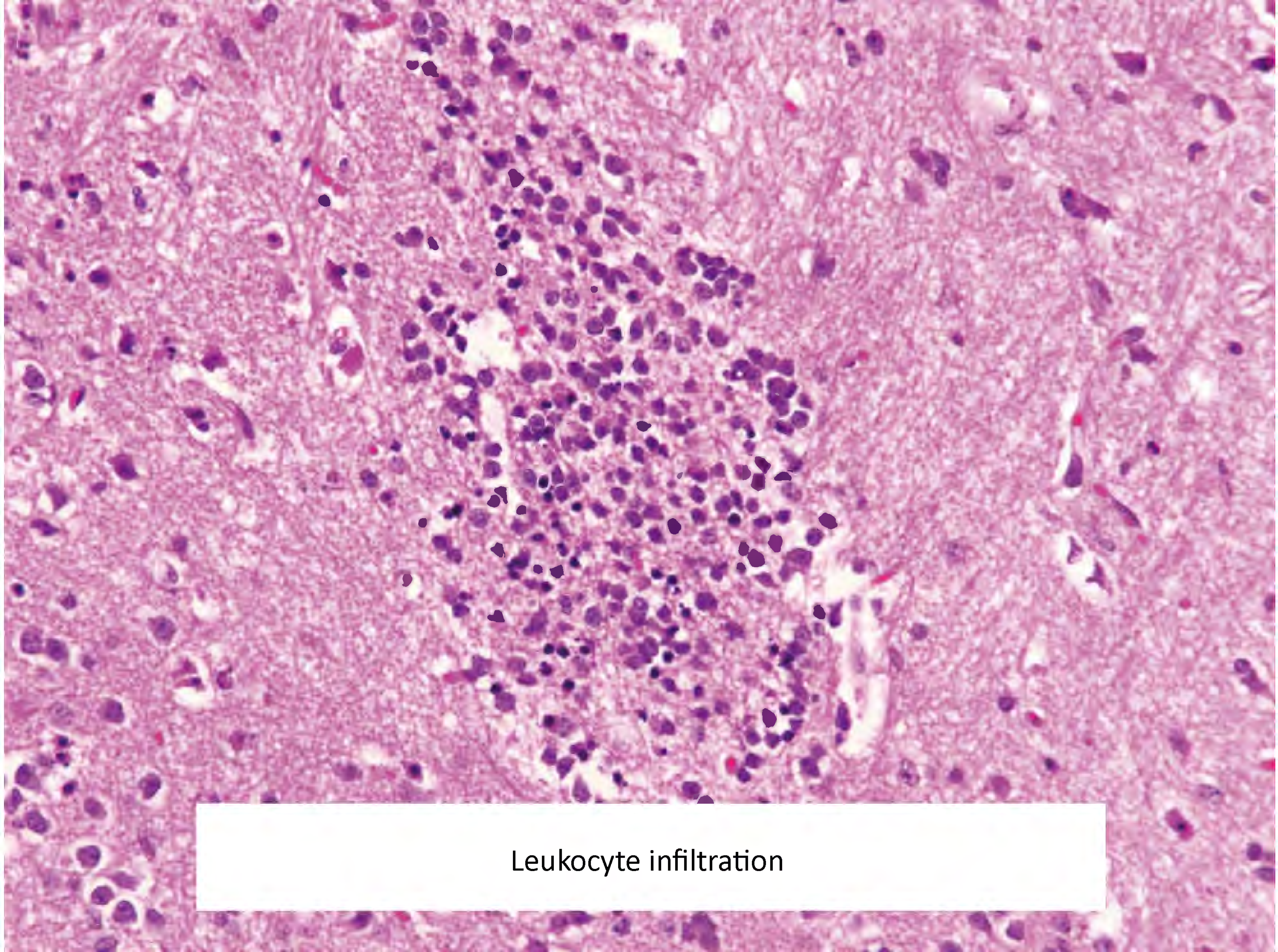

Leukocyte infiltration
