# Supplementary material for: Role of NS1 and TLR3 in Pathogenesis and Immunity of WNV
Source: Viruses. 2019 Jul 3;11(7):603. doi: 10.3390/v11070603 (PMC6669597; doi:10.3390/v11070603)
Supplement: Supplementary file 1 [file viruses-11-00603-s001.zip › Supplementary_R1/S3_FileR1.pdf]

Supplementary Data 3

Immunogenicity of gE and NS1 immunizing antigens

The ability of the gE and NS1 immunizing antigens to induce humoral immune responses in C57BL/6 mice was evaluated. An overview of the immunization schedule is given in Fig. S4A.

Briefly, two groups of 6 mice were used. Pre-immune sera were collected two days before the first round of vaccination. On day zero, 5 µg of purified antigen per mouse (either gE or NS1) was mixed with Quil-A adjuvant before being inoculated subcutaneously. Booster injections were given on day 28 and 48. Post immunization serum was obtained from each mouse on day 23, 35, and 49.

At the end of the experiment, the sera of the gE-immunized mice were tested by ELISA against gE-ELISA antigen (Fig. S4B), whereas the sera of NS1-immunized mice were tested against the NS1-ELISA antigen (Fig. S4C). The results show that all pre-immune sera did not react against the ELISA antigens, whereas it took two to three vaccinations to induce a consistent and high antibody response against the individual antigens.

It was of high interest to know, whether or not the immunizing gE antigens were at all able to induce neutralizing antibodies against WNV. Therefore, a few of the late sera were subjected to a plaque reduction neutralization test (PRNT). As shown in Fig. S4D, two out of four sera collected at day 35 from gE-immunized mice had indeed neutralizing activity against WNV. After the third immunization, indeed four out of four sera had such neutralizing activity.

Thus, these experiments showed that it took two to three vaccinations with 5 µg of our immunizing antigens to induce a reliable immune response in the vaccinated mice and, in the case of gE, to induce a reliable response of neutralizing antibodies against WNV.

Fig. S4. Immunogenicity of gE and NS1 immunizing antigens. Two groups of mice (A, B; 6 mice per group) were vaccinated using either gE- (Group A) or NS1- (Group B) immunizing antigens.

A) Immunization and bleeding schedule.
B) Development of anti-gE antibodies by ELISA (individual OD450 values shown)
C) Development of anti-NS1 antibodies by ELISA (individual OD450 values)
D) Development of WNV-neutralizing antibodies as determined by plaque reduction neutralization test (PRNT). Individual neutralizing antibody titers (log scale) are shown. The negative cut-off of this test was at a serum dilution of 1:80 (dotted line). Results from samples not exceeding this limit are kept in grey (fictional values).

Materials and Methods

Animals and immunization establishment

C57BL/6J female mice were used for animal experiments. Female wild type (WT) C57BL/6 mice (6-8 weeks old) were purchased from Charles River (Germany) and housed at the Institute of Laboratory Animal Sciences.

In the immunization experiment, 6-8 weeks old C57BL/6 mice (n=6 per group) received 5 µg gE or NS1 adjuvanted with 10 µg of Quil-A (InvivoGen, Distributor: Labforce AG, Switzerland). Two booster immunizations were given subcutaneously (s.c.) at the base of the tail on day 28 and 43 after the first immunization. Blood samples for neutralizing antibody assay and ELISA were taken at day -2, pre-immunization and day 23, 35 and 49 after primary immunization.

ELISA for gE and NS1 specific antibodies

First, gluthatione-conjucated caseine (kindly provided by Dr. Kurt Tobler, University of Zurich, Switzerland) at 1:500 in coating buffer (10 mM Na_2CO_3, 40 mM $NaHCO_3$, pH9.6) was incubated over night at 4°C in flat-bottom ELISA plates (Fisher Scientific AG, Wohlen, Switzerland). Then, excess antigen-binding sites were blocked with blocking solution (2 % milk powder in phosphate-buffered saline, supplemented with Tween 20, pH 7.4, PBST). In a next step, GST-fusion proteins (separate plates for gE and NS1, respectively) were allowed to bind as ELISA antigens to the gluthatione molecules in a native manner. For this purpose, the fusion proteins were diluted in blocking solution in a manner to provide an OD_{450} of 1.0, upon analysis with the c-myc monoclonal antibody (Life technologies, Carlsbad, CA, USA). Now, mouse sera, prediluted in blocking buffer, were incubated with the antigens. Each serum was tested in two separate dilutions, namely at 1:1000 and at 1:100. Then, goat anti-mouse-HRP-conjugated secondary antibody (GE Healthcare, Opfikon, Switzerland), diluted in blocking buffer at 1:1000 was added. Finally, TMB substrate (Thermo Scientific, Basel, Switzerland) was prepared according to the manufacturer's protocol and 100 µl were added per well. The reactions were left at room temperature and stopped after 30 minutes using an equal volume of 2 M H_2SO_4, 10 min before the absorbance was measured at 450 nm.

All volumes were 50 µL and all incubations were at 37° for one hour, except where stated otherwise. Three washings with 300 µL PBST were done between each step. The final wash before addition of the substrate solution was done with plain PBS after 5 times washing with PBST.

WNV neutralization test

Neutralizing antibodies in mouse sera were titrated by plaque reduction neutralization assay (PRNT)(L. Barzon et al., 2011). In brief, serial dilutions of each serum were mixed with 150 pfu/ml of WNV lineage 1 (Eg101 strain) and incubated for 75 min at 37°C, before inoculation onto sub-confluent monolayers of Vero cells (about 5×10^5 cells/well in 6 well plates). After adsorption for 1 h at 37°C, the inoculum was removed and the cells were overlaid with a gel made of 50% MEM 2× medium (with 4% FBS) and 50% agar noble 2% w/v in sterilized water, and incubated at 37°C, 5% CO_2. After 3 days, a secondary overlay prepared as above, but with the addition of neutral red stain, was added to the well, and on the fourth day plaques were detected as non-stained spots on the red background. The neutralizing antibody titer was calculated as the reciprocal of the serum dilution at which a 50% reduction of plaques (PRNT50) was observed compared to an inoculated control well incubated in the absence of the test serum.
