# Supplementary material for: Role of NS1 and TLR3 in Pathogenesis and Immunity of WNV
Source: Viruses. 2019 Jul 3;11(7):603. doi: 10.3390/v11070603 (PMC6669597; doi:10.3390/v11070603)
Supplement: Supplementary file 1 [file viruses-11-00603-s001.zip › Supplementary_R1/S4_FileR1.pdf]

To characterize the inflammation caused by WNV infection in the CNS, we subjected brain samples of each individual to histological examination, quantified the CNS-infiltrating neutrophils, lymphocytes, and monocytes in the infected animals by flow cytometry, and determined IFNγ and TNFα expression in the brain by RT-qPCR **Supplementary Figure S5** shows exemplary sets of Hematoxylin and Eosin (H&E) stained brain tissue sections, representing typical pictures interpreted according to increasing histopathological score.
We also assessed the infiltration of neutrophils ($CD11b^{+}Ly6G^{+}$), monocytes ($CD11b^{+}Ly6C^{high}$) and lymphocytes ($CD45^{+}CD11b^{-}$) into the infected brain by flow cytometry. The individual data are presented as an Excel sheet in **Supplementary Table 1**. Representative FACS plots showing the gating strategy for each experimental group are displayed in **Supplementary Fig. S6**. Finally, individual data for IFNγ and TNFα expression are also presented in **Supplementary Table 1**.

Supplementary Figure S5. Representative images of CNS histology in mice, showing normal brain, perivascular infiltration with individual necrotic neurons, apoptotic bodies and necrotic neurons, necrotic areas, heavy leukocytic infiltration. All pictures were taken at the same magnification and the lesions are indicated within the individual panels.

Supplementary Figure S6. Representative FACS plots showing the gating strategy. Each vaccination group is represented separately as indicated on the left. WT and TLR3KO mice are shown in different columns. $CD45^{+}$ viable single cells were analyzed for CD11b, Ly6C and Ly6G expression. Lymphocytes, neutrophils and monocytes were defined as $CD11b^{-}$, $CD11b^{+}$ $Ly6G^{+}$ $Ly6C^{lo}$, and $CD11b^{+}$ $Ly6C^{+}$ $Ly6G^{-}$, respectively. Numbers indicate the % of cells per gate in each plot.
