# Supplementary material for: Role of NS1 and TLR3 in Pathogenesis and Immunity of WNV
Source: Viruses. 2019 Jul 3;11(7):603. doi: 10.3390/v11070603 (PMC6669597; doi:10.3390/v11070603)
Supplement: Supplementary file 1 [file viruses-11-00603-s001.zip › Supplementary_R1/S1_FileR1.pdf]

# Supplementary Data 1

## Production and concentration of immunizing antigens

Recombinant bacuolviruses expressing the constructs outlined in Fig. 1 were reconstituted by transfecting Sf9 cells with the corresponding bacmid DNAs. High titer stocks of the same baculoviruses were also produced in Sf9 cells. However, to produce and purify the desired immunizing antigens, Sf9 Mimic cells were infected at high multiplicity. Seven days post inoculation, the cells were harvested and lysed as described in Materials and Methods and the desired fusion proteins were enriched by using their 6xhis tail binding to Talon resins charged with cobalt, before eluting with imidazole (see Materials and Methods). The desired fusion proteins were detected on Western blots by using a monoclonal antibody against the V5-tag. As shown in Fig. S1A, a single band was detected by the anti-V5 mcAb in the original lysate (starting material, lane 1) of the baculovirus expressing the gE-V5-6xhis fusion protein. The detected fusion protein migrated just above the 53 kDa marker, which fitted well with the predicted molecular weight (55 kDa) of the desired gE-fusion protein. Only shadows of the same protein were detected in the flow through fraction (lane 2) and in the first eluate (lane 3). However, a much more concentrated band of the same apparent mobility appeared in the second elution fraction (lane 4) and again a fainter band in the third elution fraction (lane 5). These observations suggested that the desired WNV gE-fragment accumulated in elution fraction 2, which was subsequently used as the source for gE-immunizing antigen. Essentially the same approach was used for producing and concentrating the NS1 fusion protein. The corresponding recombinant baculovirus was reconstituted and expanded in Sf9 cells before antigen production was done in Sf9 Mimic cells. The corresponding results are shown in Fig. S1B. In the starting material, a relatively fat band was detected migrating at around 48 kDa, which most probably represented the non-glycosylated precursor of the NS1-fusion protein. An additional, somewhat fainter and less sharp band migrated slightly slower and was considered to represent the glycosylated NS1-fusion protein. Minor amounts of the same bands were detectable in the elution fractions 1 and 2, whereas a much-concentrated double band was present in elution fraction 3. These observations suggested that the desired WNV NS1-fragment accumulated predominantly in elution fraction 3, which was subsequently used as the source for NS1-immunizing antigen.
Together, these observations indicated that recombinant baculoviruses had been generated, from which partially purified and enriched WNV gE- and NS1-immunizing antigens could be produced.

**Fig. S1. Enrichment of recombinant WNV proteins from lysates of baculovirus-infected insect cells.** Mimic Sf9 cells were inoculated with recombinant baculoviruses expressing WNV gE (A) or WNV NS1 (B). Seven days post inoculation, the cells were harvested and resuspended in lysis buffer (see Materials and Methods). For enrichment, soluble 6xhis- and V5-tagged proteins were allowed to bind to Talon resins charged with cobalt. After washing, the desired proteins were eluted by adding 200 mM imidazole. Samples from starting materials (SM), non-bound flow through (FT, only in A) three consecutive elutions (E1, E2, E3) were loaded on SDS polyacrylamide gels, electrophoresed, and transferred to PVDF membranes before staining with a monoclonal antibody against the V5 epitope. The expected molecular weight of the gE-fragment was 53 kDa; that of the NS1-fragment was 48 kDa. (Molecular weight marker: PageRuler Plus prestained protein ladder)

## ELISA antigens

To measure antibody development upon the vaccination of mice with the above immunizing antigens, ELISA antigens were also produced in recombinant baculoviruses. However, the targeted sequences were shortened and different tags were used (see Materials and Methods and also Fig. 1B, 1C). The gE-DIII protein was efficiently secreted to the cell culture supernatant and could be acquired directly from the cell culture medium.

However, the NS1-dC protein remained predominantly cell-associated and was extracted from the inoculated SF9 cells (see Materials and Methods).

Samples of the desired proteins were detected by western blot using an anti-myc monoclonal antibody (mcAb)(Fig. S2A). The gE-DIII protein migrated at its predicted molecular weight of 46 kDa, whereas the NS1-dC had an apparent mobility of approximately 58 kDa. Thus, these fusion proteins met our expectations concerning their molecular weights and could be detected by anti-c-myc mcAb.

In order to test their ability of binding to glutathione via their GST-tags, ELISA plates were coated with glutathione-conjugated casein before serial dilutions of the fusion proteins were added to separate wells. Each antigen dilution was tested in duplicate. After washing, glutathione-bound protein was quantitatively detected by ELISA using the anti-c-myc mcAb (Fig. S2B). In the case of gE-DIII-c-myc-GST, maximum binding was detected until a dilution of 1:16, after which the protein diluted out to almost background levels until dilution of 1:512. At 30 minutes after addition of the substrate, an optical density (OD at 450 nm) of approximately 1.0 was achieved with an antigen dilution of 1:128. The NS1-dC-c-myc-GST antigen was present at a lesser abundance, providing saturation of the coated glutathione up to a dilution of 1:4, from where it diluted out in a regular fashion. OD 1.0 was observed at a dilution of 1:32.

Since each detected molecule comprised one c-myc as well as one GST epitope, we considered equal optical density to represent equal molarity of bound protein. Together, these results provided evidence that our newly produced antigens were able to bind quantitatively and in an equimolar fashion to glutathione-conjugated casein on ELISA plates. Thus, these fusion proteins were ready to be used in ELISAs for detection of antibodies against either WNV gE or WNV NS1.

**Fig. S2. Characterisation of ELISA antigens**. A) Sf9 cells were inoculated with recombinant baculoviruses expressing either the DIII domain of WNV gE (lane 1) or the exodomain of WNV NS1 (lane 2). Both constructs were made to comprise a C-terminal c-myc tag and GST-sequences. Samples of each ELISA antigen (see Materials and Methods) were loaded on SDS polyacrylamide gels, electrophoresed, and transferred to nitrocellulose membranes before staining with a monoclonal antibody against the c-myc epitope. The expected molecular weight of the gE-DIII-c-myc-GST fusion protein was 46 kDa; that of NS1-dC-c-myc-GST was 58 kDa (Molecular weight marker: Pageruler Plus prestained protein ladder).

B) ELISA plates were coated with glutathione-conjugated casein, before two-fold serial dilutions of gE-DIII-c-myc-GST (left panel) or NS1-dC-c-myc-GST (right panel) were added. GST-bound fusion proteins were detected with anti-c-myc mcAb and goat-anti-mouse-peroxidase. 30 min after addition of TMB substrate, the OD values were measured at 450 nm.

## Materials and Methods

Cell lines

*Spodoptera frugiperda*-derived Sf9 and Mimic Sf9 were obtained from Thermo Fisher Scientific (Reinach, BL, Switzerland) and maintained as monolayers at 27°C. Conventional Sf9 cells were cultured in Grace's insect medium supplemented with 10% FCS and used for transfections, viral stock production, and ELISA antigen production. The Mimic SF9 cells were cultured in serum-free SF900 III medium (Thermo Fisher Scientific) and used for expression of immunizing antigens.

**Baculovirus production using SF9 cells**

In order to reconstitute infectious baculovirus stocks, 2 x $10^5$ SF9 cells per well were seeded into 24-well plates before being transfected with bacmid DNA in 3:1 ratio using Fugene HD (Promega AG, Duebendorf, Switzerland) according to the manufacturer's protocol. P0 stocks were collected after 5 days. 100 µl of P0 stocks were used to produce the P1 stocks on a 25 $cm^2$ flask plated with 4 x $10^6$ SF9 cells. For the P2 stocks, 12 x $10^6$ SF9 cells were plated on a 75 $cm^2$ flask and 100 µl of P1 stock was added to the medium. The viral titers for the P2 stocks were determined using an end-point dilution assay in which the protein tag (either V5 or c-myc) was targeted with commercially available monoclonal antibodies (Thermo Fisher Scientific). The reactions were visualized as described in the Western blot protocol below. After titration, the P2 stocks were used for high multiplicity inoculations (MOI=4) towards large scale protein production.

**Immunizing antigen production and enrichment in Mimic SF9 cells**

6 x $10^7$ Mimic SF9 cells were infected with P2 viral stocks of gE or NS1 at a multiplicity of infection (MOI) of 4. The cells were incubated for 7 days at 27°C. After 7 days, cells were collected, centrifuged at 3000 x g for 15 minutes in Heraeus, Multifuge 3S-R centrifuge. Since preliminary experiments had shown that most of the recombinant proteins were retained inside of the Sf9 cells, the supernatant was discarded and the cell pellets were resuspended in 8 ml of lysis buffer (50 mM $NaH_2PO_4$, 300 mM NaCl, 8 M Urea, 1 mM $MgCl_2$, pH 8.0, supplemented with protease inhibitor cocktail (Roche, Basel, Switzerland) and BitNuclease (BioTool AG, Kirchberg, Switzerland)). This lysate was equilibrated at room temperature for 30 minutes before being subjected to sonication with 2 shorts bursts of 30 seconds each. The lysate was then centrifuged at 3000 x g for 15 minutes at 4°C and carefully transferred to a new tube. His-tagged proteins were purified using the Talon metal affinity resin (Clontech, Takara Bio Europe, Saint-Germain-en-Laye, France) charged with Cobalt ions using the gravity flow columns. Resin equilibration was performed according to the manufacturer's protocol before 80 µL of the resin slurry was transferred to the cell lysate and gently rotated overnight at 4°C to allow the binding of the protein to the resin. The cell lysate-resin suspension was centrifuged at 750 x g for 5 minutes in Heraeus Multifuge 3S-R centrifuge and washing was carried out as per the protocol. Wash buffer constituted of 50 mM $NaH_2PO_4$, 300 mM NaCl, 8 M Urea with 5 mM imidazole, pH 7.0. Proteins were eluted in 200 µL of elution buffer (50 mM $NaH_2PO_4$, 300 mM NaCl, 8 M Urea with 200 mM imidazole, pH 7.0). Protein expression and enrichment was confirmed by Western Blot analysis.

**Western Blot**

Standard methods were used to produce protein extracts, separate them electrophoretically on 10-12.5% SDS polyacrylamide gels, transfer to PVDF membranes, and stain with mouse monoclonal antibodies (either anti-V5 or anti-c myc at a dilution of 1:5000; Thermofisher Scientific, Basel, Switzerland) followed by biotinylated horse anti-mouse-IgG serum (1:500; Vector laboratories, Peterborough, United Kingdom) and Vectastain's peroxidase ABC

solution (Sigma, Buchs, Switzerland). Lastly, 3, 3' – Diaminobenzidine tetrahydrochloride substrate (Sigma) was used for visualization of the bands.

**ELISA antigen production**

ELISA antigens were produced in SF9 cells. The cell culture medium from P2 stock production of gE.DIII-c-myc-GST, as previously described, with protease inhibitor was used as the antigen for ELISA antibody detection. However, NS1-dC-c-myc-GST protein remained predominantly within the cell and was extracted from the cell pellet. Cell pellet was obtained from the P2 stock production and centrifuged at 3000 x g for 15 mins. The supernatant was discarded and the cell pellet was resuspended in RIPA buffer (50 mM Tris-HCl, pH 7.4, 1% NP40, 0.25% Sodium Deoxycholate, 150 mM NaCl with protease inhibitor (Roche, Basel, Switzerland)). The cell suspension was sonicated twice for 30 seconds, centrifuged at 3000 x g for 15 minutes at 4°C. The supernatant/soluble fraction was used for detection of anti-NS1 antibodies in the ELISA.

**ELISA establishment using the GST-c-myc tagged antigens**

Wells were coated overnight at 4°C with 50 µl linked casein-glutathione, diluted 1 in 500 in coating buffer (10 mM $Na_2CO_3$, 40 mM $NaHCO_3$) as established in-house. The wells were washed thrice with 300 µl PBST (0.3 % Tween-20 in PBS) and blocked with 300 µl of blocking solution (2% milk powder in PBST) per well and incubated at 37°C for an hour. A serial dilution of the GST-tagged gE (cell medium supernatant containing non-purified gE protein) or NS1 (cell lysate in RIPA buffer) was done and 50 µl/well of each dilution was added and the plate was incubated at 37°C for another hour and then washed thrice with 300 µl/well of PBST. 50 µl of anti- c-myc mouse monoclonal antibody (1:1000) was added per well and incubated for one hour at 37°C. Plate was washed again 3x with PBST. After washing, 50 µl of goat anti mouse HRP conjugated secondary antibody (GE Healthcare) (1:1000, in blocking buffer) was added and incubated for another hour at 37°C. Plate was then washed 5 x with 300 µl PBST and once with PBS to get rid of Tween-20. TMB substrate (Thermo Scientific) was prepared as per the manufacturer's protocol and 100 µl was added per well. Reaction was stopped after 30 minutes using 2M $H_2SO_4$ and absorbance was measured after 10 minutes at 450 nm.
