# Supplementary figures and images for: Role of NS1 and TLR3 in Pathogenesis and Immunity of WNV
Source: Viruses. 2019 Jul 3;11(7):603. doi: 10.3390/v11070603 (PMC6669597; doi:10.3390/v11070603)

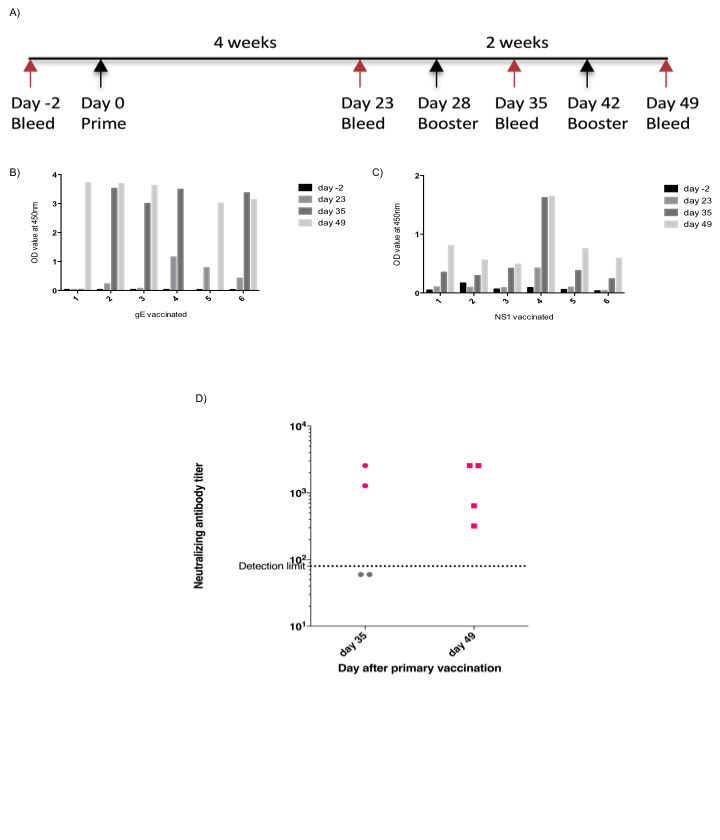

Supplement: Supplementary file 1 [file viruses-11-00603-s001.zip › Supplementary_R1/Fig S4.tif]

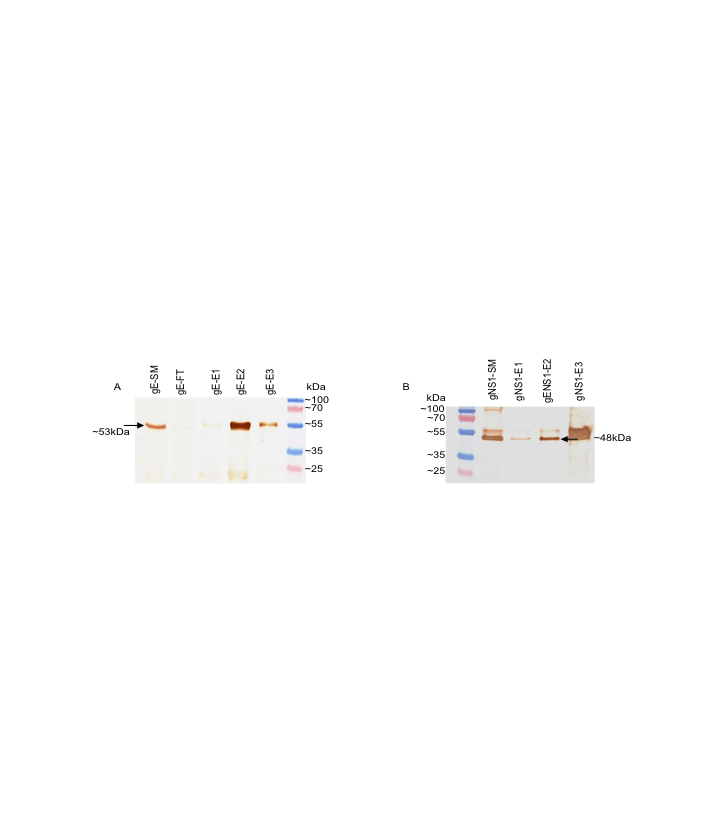

Supplement: Supplementary file 1 [file viruses-11-00603-s001.zip › Supplementary_R1/Fig S1.tif]

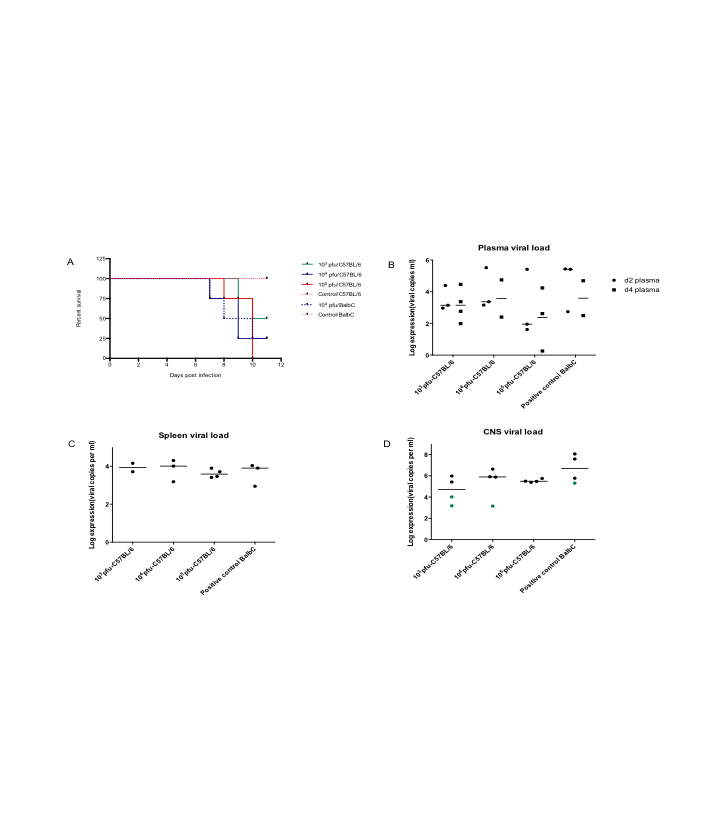

Supplement: Supplementary file 1 [file viruses-11-00603-s001.zip › Supplementary_R1/Fig S3.tif]

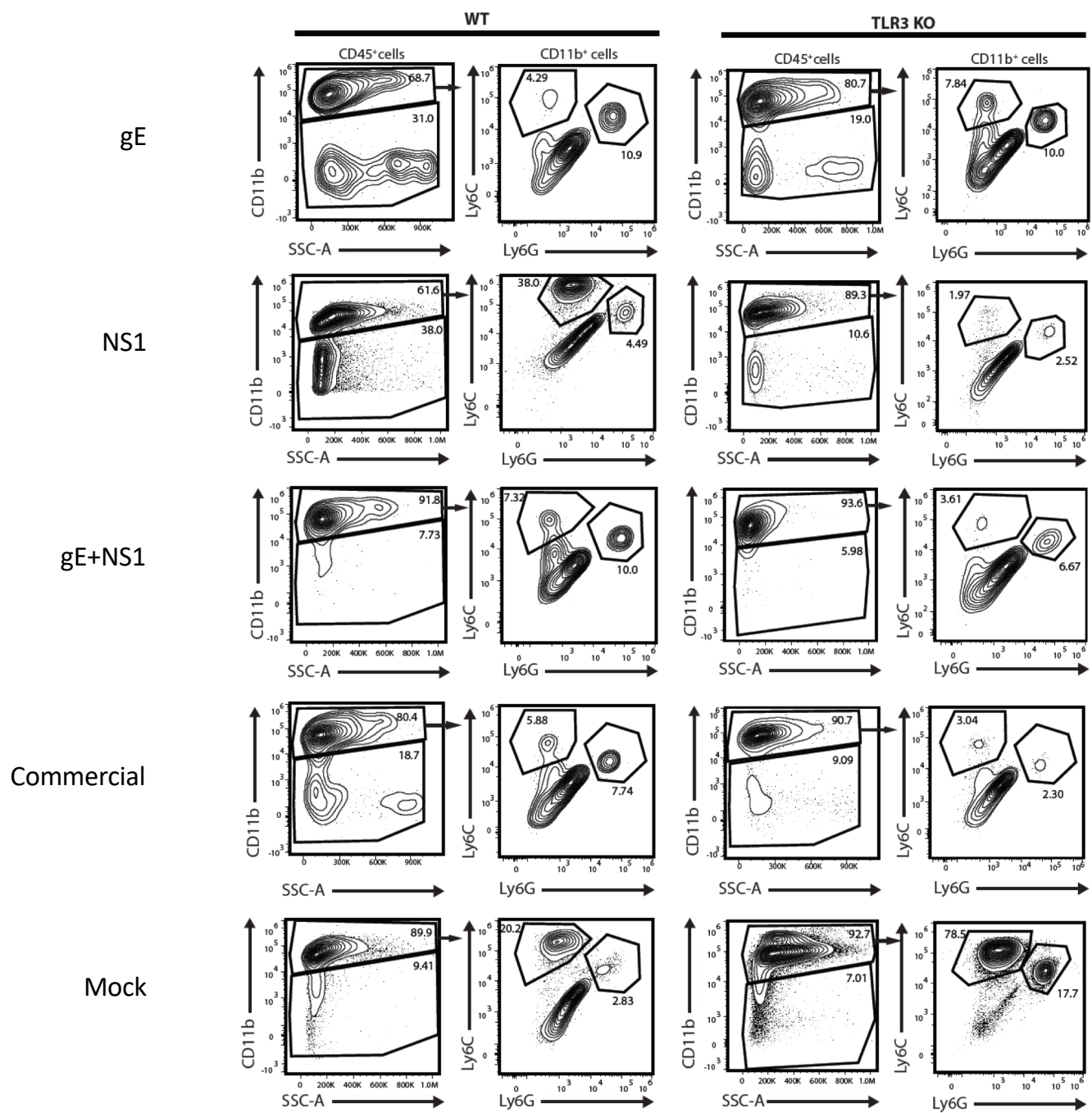

Supplement: Supplementary file 1 [file viruses-11-00603-s001.zip › Supplementary_R1/Fig S6R1.pdf]

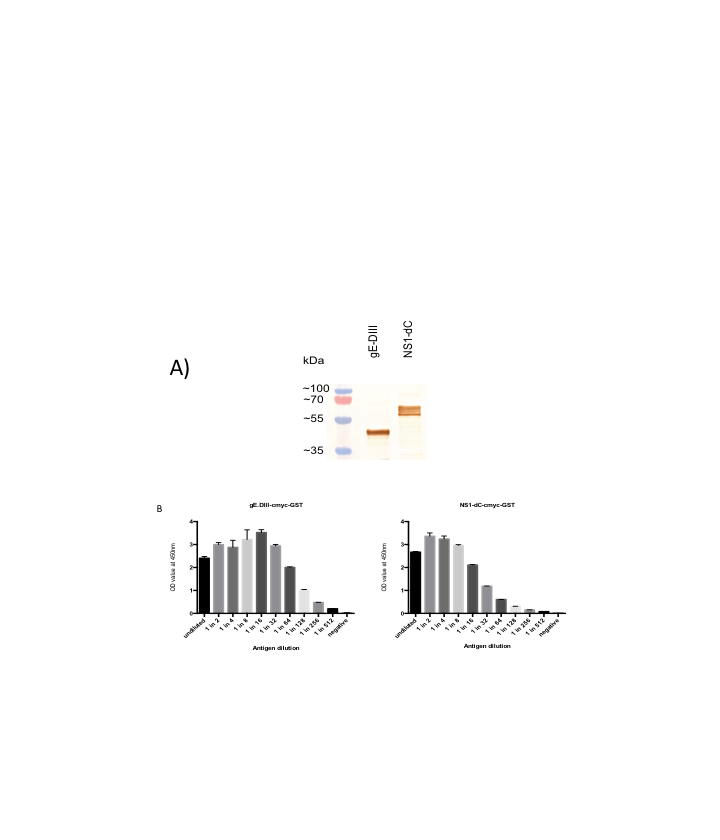

Supplement: Supplementary file 1 [file viruses-11-00603-s001.zip › Supplementary_R1/Fig S2.tif]
